# Fractional Doses of Pneumococcal Conjugate Vaccine: A Non-Inferiority Trial

**DOI:** 10.1056/NEJMoa2314620

**Published:** 2024-09-26

**Authors:** Katherine E. Gallagher, Ruth Lucinde, Christian Bottomley, Mary Kaniu, Badaud Suaad, Mary Mutahi, Laura Mwalekwa, Sarah Ragab, Louise Twi-Yeboah, James A. Berkley, Mainga Hamaluba, Angela Karani, Jimmy Shangala, Mark Otiende, Elizabeth Gardiner, Daisy Mugo, Peter G. Smith, Collins Tabu, Fred Were, David Goldblatt, J. Anthony G. Scott

**Affiliations:** 1 Faculty of Epidemiology and Population Health, London School of Hygiene and Tropical Medicine, UK; 2 KEMRI-Wellcome Trust Research Programme, Kilifi, Kenya; 3 Department of Paediatrics, Coast General Teaching & Referral Hospital, Mombasa, Kenya; 4 Great Ormond Street Institute of Child Health, University College London, UK; 5 Centre for Tropical Medicine & Global Health, University of Oxford, UK; 6 Immunization, UNICEF, Nairobi; 7 School of Medicine, University of Nairobi

## Abstract

**Background:**

Pneumococcal conjugate vaccines (PCVs) are an expensive component of the routine immunisation schedule. We assessed whether immunogenicity was non-inferior after fractional doses of PCV10 (GlaxoSmithKline plc.) or PCV13 (Pfizer Inc.), when compared to full doses, and analysed vaccine-serotype carriage prevalence.

**Methods:**

2100 healthy infants were enrolled and randomised into seven equal-sized trial arms. Doses were delivered in the 2 prime + 1 boost (2p+1) schedule in six trial arms: A) Full dose PCV13, B) 40%-PCV13, C) 20%-PCV13, D) Full dose PCV10, E) 40%-PCV10, F) 20%-PCV10. Participants in a seventh trial arm received full dose PCV10 in a 3p+0 schedule. Immunogenicity was assessed 4-weeks post-prime and 4-weeks post-boost. At 4-weeks post-prime, non-inferiority was declared if the difference in the proportion of ‘responders’ was not more than 10%. At 4-weeks post-boost, non-inferiority was declared if the ratio of the geometric mean concentration (GMC) of IgG was more than 0.5. Carriage was assessed at 9 and 18 months of age.

**Results:**

In the per-protocol analysis, 40%-PCV13 met the non-inferiority criteria for 12/13 serotypes post-prime and 13/13 serotypes post-boost. The 20%-PCV13, 40%-PCV10 and 20%-PCV10 arms elicited inferior immunogenicity to the full dose comparators. Vaccine-type carriage prevalence was similar across the PCV13 arms at 9 and 18 months of age.

**Conclusions:**

A 3-dose schedule of using 40% of the standard dose of PCV13 was non-inferior to the standard dose after boosting for all included serotypes but lower doses of PCV13 and PCV10 were not non-inferior. (Trial Registration: ClinicalTrials.gov ID: NCT03489018; Pan African Clinical Trial Registry ID: PACTR202104717648755.)

Pneumococcal conjugate vaccines (PCVs) have proven to be highly effective in reducing vaccine-type pneumococcal disease^1–3^. Since 2010, Gavi, the Vaccine Alliance, has funded PCV introduction in 47 ‘Gavi-eligible’ low and lower-middle-income countries (LMICs). However, even at a subsidised cost of US$2.00-3.30 per dose, the PCV programme is the most expensive component of the routine immunisation schedule in many LMICs^4^. The World Health Organization (WHO) recommends a primary series at 6-8, 10-12 and 14-20 weeks of age (the ‘3p+0’ schedule), or two primary doses at 6-8 and 14-16 weeks of age with a booster at least 6 months after the 2^nd^ dose (the ‘2p+1’ schedule)^5^. The sustainability of the PCV programme is of concern in countries that are transitioning out of Gavi support and taking on the full cost of procuring the vaccine. Furthermore, for middle-income countries ineligible for Gavi support, a reduction in the cost of PCV may enable vaccine introduction where it is currently unaffordable.

Fractional doses of antigen have been shown to induce non-inferior immune responses to full doses in trials of vaccines against *Haemophilus influenzae* type b (Hib)^6–10^, *Neisseria meningitidis*^11^, Yellow Fever^12–15^ and Polio^16–20^. A systematic review^21^ identified one early trial of a pentavalent PCV that documented serotype-specific immune responses that reached the threshold of protection (≥0.35 mcg/ml; established following later efficacy trials) after a dose of just 0.5 mcg of antigen, without an adjuvant. This dose equates to 23% of the current dose in Prevnar13, PCV13 (Pfizer Inc.), and 50% of the dose in Synflorix, PCV10 (GlaxoSmithKline, GSK, Plc.; Table 1)^22^, though the two vaccines use different carrier proteins and conjugation methods^23,24^.

We aimed to assess whether the serotype-specific immunogenicity of fractional doses (20% or 40%) of PCV10 or PCV13, administered in a 2p+1 schedule, was non-inferior to the immunogenicity of full doses. We also aimed to also assess the prevalence of vaccine-type carriage.

## Methods

### The study population

The trial enrolled children at 9 health facilities in Kilifi and Mombasa Counties, Kenya. PCV10 (GSK) was introduced in the national EPI programme in Kenya in 2011 in a 3p+0 schedule. In 2017, among children aged 12-23 months, coverage of the third dose was 89%^25^. In 2016, there were 3.2 cases of vaccine-type invasive pneumococcal disease (IPD) per 100,000 person years in children aged <5 years^26^.

### Trial design

**Figure 1:**
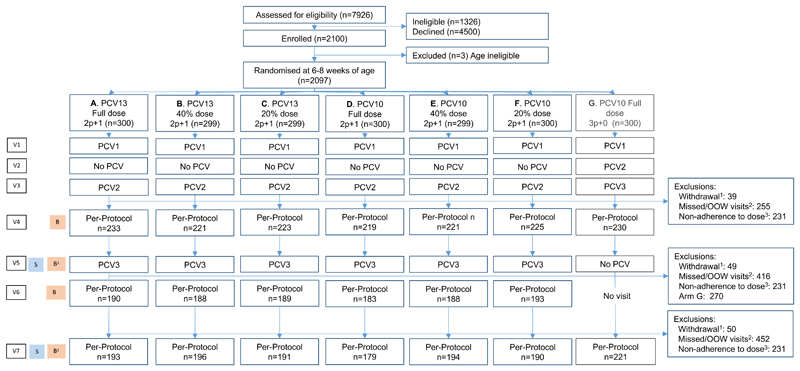
The clinical trial flow ^1^ Withdrawals include parental withdrawals, investigator-initiated withdrawals, deaths and 3 participants who were identified as ineligible for the study after randomization; ^2^ Missed or out of window visits refer to missed or out of window vaccination or sampling visits, and missed samples; ^3^ Non-adherence to dose includes participants who received full doses outside of the study activities (predominantly during the pause in research activities during the COVID-19 pandemic), and 13 randomization and vaccination errors. Abbreviations: B: Blood sample (Half of the participants in Arms A-F were randomly assigned to blood draws at V5 and V7); OOW: out-of-window (visit 4 and visit 6 had to occur 28 days after the last PCV dose, all visit windows were +/- 7days apart from visit 5 which could occur between D214 and D312, and visit 7 (+/- 14 days)); PCV: Pneumococcal Conjugate Vaccine (doses 1-3); S: Nasopharyngeal swab; V1: visit 1 at Day 0; V2: visit 2 at Day 28 +/- 7 days; V3: visit 3 at Day 56 +/-7 days; V4: visit 4 at 28 days post-V3 +/- 7 days; V5: visit 5 at Day 228 +3months/-2weeks; V6: visit 6 at 28 days post-V5 +/- 7days; V7: visit 7 at Day 502 +/- 14 days.

Anticipating a potential change in the schedule, we designed the study to deliver PCV in a 2p+1 schedule with primary doses at 6 and 14 weeks of age and a booster dose with MCV1 at 9 months of age. MCV1 coverage was approximately 78% in 2017^27^. At 6-8 weeks of age, 2100 infants were randomised equally into seven trial arms and followed until 18 months of age. Six trial arms delivered PCV in a 2p+1 schedule: Arm A) Full dose PCV13 B) 40%-dose PCV13 C) 20%-dose PCV13 D) Full dose PCV10 E) 40%-dose PCV10 F) 20%-dose PCV10. The seventh trial arm, Arm G, delivered PCV10 in a 3p+0 schedule to bridge the findings to the existing dose and schedule in Kenya (Figure 1). Details of the study design can be found in the protocol and SAP at nejm.org.

### Study procedures

Community Health Volunteers identified households with new-borns and invited the carers to the health facility to provide information about the study. We recruited any healthy infant (i.e. no acute febrile illness on the day of enrolment) aged 6-8 weeks, who was eligible for vaccination in the routine immunisation programme but had not yet received their first dose of PCV. Infants more than 8 weeks of age were excluded from the trial. Less than 5% of mothers were HIV infected (Suppl. Table 2B) and infants were enrolled irrespective of HIV status.

Each infant was randomly allocated to one of the seven trial arms with equal probability using sequentially numbered, sealed envelopes. Computer-generated randomisation codes were prepared in advance by an independent statistician using block sizes of 14. Parents of participants in arms A-F were blinded to the dose allocated. Other than the team administering vaccine, all other study personnel were blinded to allocation of participants until the end of the study. Blinding of arm G, was not possible. A research nurse prepared 0.5ml as a full dose, 0.2ml as a 40% dose or 0.1ml as a 20% dose and administered the vaccine in a masked syringe, intramuscularly in the right anterolateral thigh muscle. Participants received other immunisations according to the routine schedule.

At 28 days (visit 2) and 56 days (visit 3) post-enrolment, the primary series of PCV was administered according to the child’s allocated schedule. Four weeks after visit 3, at approximately 18 weeks of age, a blood sample was collected from all infants. At approximately 9 months of age (visit 5) a single nasopharyngeal swab was collected from all participants and infants in arms A-F received their third, booster dose of PCV. Four weeks after the booster dose (visit 6), a post-boost blood sample was collected from all participants in arms A-F. Finally, at approximately 18 months of age (visit 7), a nasopharyngeal swab was taken from all participants. A blinded member of the study team assessed each child 7 days after each PCV dose for injection site abscesses.

Adverse events (AEs) and serious adverse events (SAEs) were defined in accordance with the International Conference on Harmonization (ICH) Guidelines for Good Clinical Practice. AEs were treated by the unblinded study nurses stationed at each facility. All SAEs were treated by a blinded study clinician at hospital.

### Laboratory methods

A maximum of 2ml of whole blood was collected via venepuncture and transported to the KEMRI-Wellcome Trust Research Programme (KWTRP) laboratory at 2-8 degrees C; serum was separated within 48 hours. At the WHO Reference Laboratory for Pneumococcal Serology based at the Great Ormond Street Institute of Child Health, sera were assayed for IgG to vaccine-type capsular polysaccharides using an ELISA (http://www.vaccine.uab.edu/ELISA%20Protocol.pdf) and, on a subset (n=50, 1-month post-boost), for functional antibody using the Multiplexed Opsonophagocytic Assay (MOPA; http://www.vaccine.uab.edu/UAB-MOPA.pdf)^28^. Samples were analysed for IgG to vaccine-typess, except for samples from the routine immunisation arm (PCV10 3p+0) which were assayed for 7 vaccine-typess due to funding constraints.

A single nasopharyngeal swab was taken at 9 and 18 months of age and transported in 1ml skimmed-milk-tryptone-glucose-glycerin (STGG) transport medium to the KWTRP laboratories for processing using standard methods^29,30^. A primary culture was prepared on blood agar with gentamicin, one colony on the plate was selected at random for serotyping by latex agglutination and confirmatory Quellung reaction. Polymerase Chain Reaction (PCR) was performed for quality control purposes on 10% of the samples and as a confirmatory test for samples that had ambiguous or negative Quellung tests. Vaccine-type carriage was defined if a vaccine serotype was identified by latex agglutination and confirmatory Quellung reaction^30^.

### Statistical analyses

The statistical analysis plan prespecified that non-inferiority would be determined using the lower limit of a one-sided 95% confidence interval (i.e. equivalent to a 2-sided 90%CI). The analyses presented in this paper use more-stringent, 2-sided 95%CIs in conformity with the non-inferiority analyses used to licence PCV13^31,32^. At 4-weeks post-boost, non-inferiority was declared if the lower limit of the 95% CI for the ratio of the geometric mean concentration (GMC) of IgG (fractional/full dose arms) was more than 0.5. At 4 weeks after the primary series (18 weeks of age), ‘responders’ were defined as those with serotype-specific IgG antibody concentration ≥0.35 mcg/ml^33^; non-inferiority was declared if the lower limit of the 95% CI around the difference (fractional dose group – full dose group) in the proportion of ‘responders’ was >-10%^31^. *A priori*, a vaccine dose was defined as non-inferior if it met the non-inferiority criteria for at least 8 of the 10 vaccine types in the PCV10 arms or at least 10 of the 13 vaccine types in the PCV13 arms.

Analyses were restricted to the per-protocol population; this comprised randomized participants in arms A-G who had completed their allocated schedule with their allocated vaccine dose and had at least one blood sample within window (for immunogenicity analyses) or at least one carriage sample within window (for carriage analyses). For the non-inferiority analyses of immunogenicity, the widths of the intervals have not been adjusted for multiplicity and should not be used in place of hypothesis testing. For the secondary analysis of carriage, the widths of the confidence intervals around the risk differences have not been adjusted for multiplicity and should not be used in place of hypothesis testing.

The required sample size for the trial was calculated to ensure sufficient power for both the post-prime and post-boost non-inferiority analyses^34^. To declare non-inferiority at the post-prime timepoint with 90% power, we estimated that we would need to enrol 300 infants per arm, assuming serotype-specific response rates^35–39^ and 5% loss to follow up. This sample size conferred >99% power to declare non-inferiority in the analyses post-boost, assuming similar GMCs to those in South African children^39^.

Approvals were obtained from the Kenyan Medical Research Institute Scientific & Ethics Review Unit (SERU) and the London School of Hygiene & Tropical Medicine (LSHTM) Ethics Committee. Written informed consent was obtained from at least one caregiver of all infants enrolled in the study. An independent data and safety monitoring committee provided trial oversight. ClinicalTrials.gov ID: NCT03489018.

## Results

Between March 2019 and November 2021, 2100 infants and their parents were enrolled into the trial; 673 (32%) participants were enrolled by March 2020, trial activities were then paused due to the COVID-19 pandemic; 1427 infants were enrolled between October 2020 and November 2021 (Figure 1). Due to the disruption of follow-up in 2020, 1572 participants out of 2100 (75%) were included in the per-protocol analysis at 18 weeks of age. For the post-boost immunogenicity analysis, 1131 (63%) participants of the 1797 who were allocated to 2p+1 arms were included in the per-protocol analysis (Suppl. Tables 1A-B). For the carriage prevalence analyses, 1439 participants (69%) were included in the per-protocol analysis at 9 months of age and 1364 (65%) at 18 months of age (Suppl. Tables 1C-D). The characteristics of participants in the per-protocol analysis were balanced across the arms with respect to sex, HIV exposure (maternal HIV status), infant weight at enrolment, breastfeeding at 10 months of age and the timing of their boost dose (Suppl. Tables 2A-D). The per-protocol population at 10-months of age comprised a representative distribution of infants by sex and ethnic group, compared to the Kilifi population^25,40^ (Suppl. Table 2E).

### Immunogenicity post-primary series

**Figure 2:**
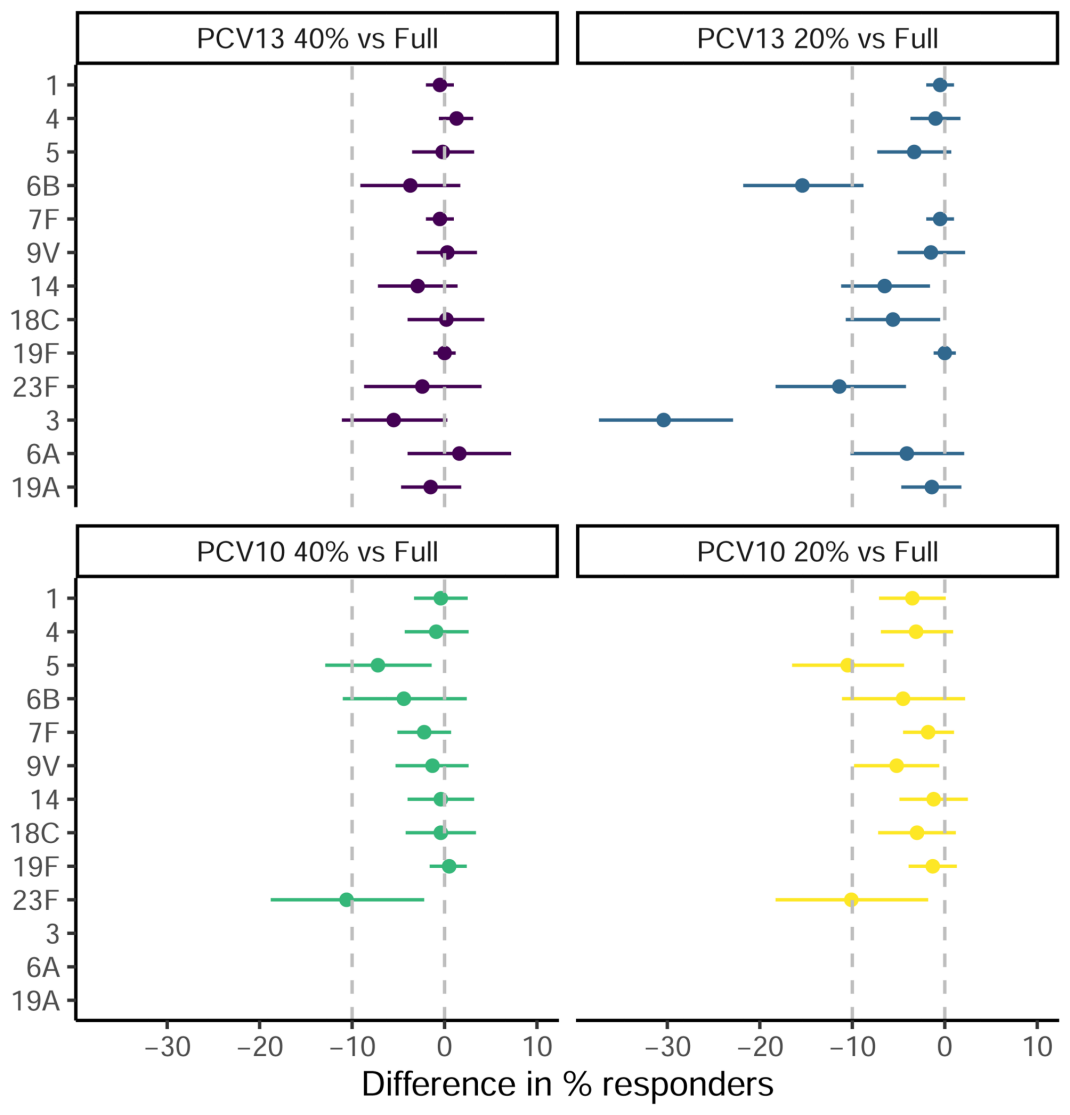
The difference in the proportion of responders (full dose-fractional dose, 95%CI) at 4-weeks post primary series (18 weeks of age) Notes: Differences are displayed with 95%CI as per Suppl Table 3B; the widths of the intervals have not been adjusted for multiplicity and should not be used in place of hypothesis testing.

Compared to 2 full doses of PCV13, the non-inferiority criterion was met for 12 of the 13 serotypes in the 40% PCV13 recipients, but for only 7 of the 13 serotypes in the 20% PCV13 recipients. Compared to 2 full doses of PCV10, the non-inferiority criterion was met for 7 of the 10 serotypes in the 40% PCV10 recipients, and only 7 of the 10 STs in the 20% PCV10 recipients (Figure 2). A primary series of 2 PCV10 doses was non-inferior to a primary series of 3 PCV10 doses for 6 of the 7 serotypes assayed (Suppl. Tables 3A-C).

### Immunogenicity post-boost

**Figure 3:**
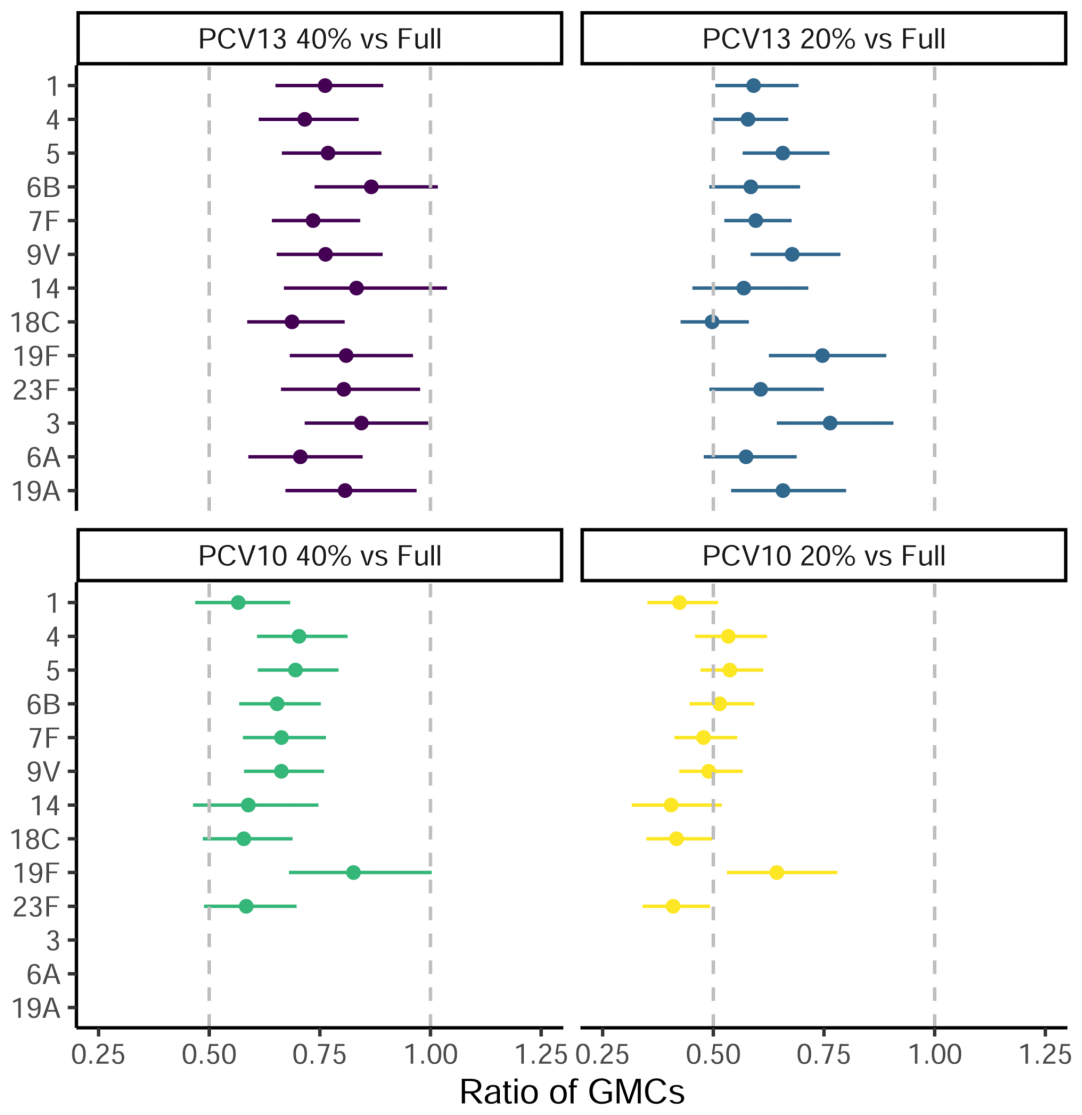
The ratio of GMCs post-boost (full dose: fractional dose, 95%CI; approximately 10 months of age) Notes: Ratios are displayed with 95%CI as per Suppl. Table 4B, the widths of the intervals have not been adjusted for multiplicity and should not be used in place of hypothesis testing

In the 2p+1 arms, compared to full dose PCV13 recipients, the post-boost non-inferiority criterion was met for 13 of the 13 serotypes in the 40% PCV13 recipients, but for only 6 of the 13 serotypes in the 20% PCV13 recipients. In the 2p+1 arms, compared to full dose PCV10 recipients, the post-boost non-inferiority criterion was only met for 6 of the 10 serotypes in the 40% PCV10 recipients and only 1 of the 10 STs in the 20% PCV10 recipients (Figure 3; Suppl. Tables 4A-C). In the sub-set of participants who had a sample assayed for opsonophagocytic function, the proportion of samples with geometric mean OPA titres of >8 was high across all arms and serotypes (suppl. Tables 4D-F).

### Carriage prevalence

At 9 months of age, PCV10-type carriage prevalence varied between 4.4% and 8.6%, across the 7 trial arms. At 18 months of age, 16-19% of participants in the PCV13 arms carried PCV13-types; 3-11% of the 2p+1 PCV10 recipients carried PCV10-types. At 18 months of age, 9.5% of the full dose PCV10 3p+0 arm carried PCV10-types (Table 2; Suppl. Tables 5).

### Safety

No injection site abscesses were recorded. A total of 61 cases of non-severe pneumonia and 65 SAEs were recorded; these were distributed across the arms evenly (Suppl. Table 6).

## Discussion

A 2p+1 schedule of 40% doses of PCV13 elicited non-inferior IgG responses after both the primary series and the booster dose, when compared to a full dose 2p+1 schedule at peak immune response time points. Smaller, 20%, doses of PCV13 elicited inferior immunogenicity, when compared to a full dose schedule. Vaccine-type carriage prevalence was similar across the PCV13 arms at 9 and 18 months of age. The per-protocol populations for the non-inferiority analyses were smaller than planned, which is likely to have reduced the precision with which we could estimate the ratios of the proportion of responders and of GMCs. The 20% PCV13 arm narrowly missed the non-inferiority criteria for some serotypes and may have met the criteria with a larger sample size.

Fractional dose schedules of PCV10 failed to meet non-inferiority criteria for immunogenicity and demonstrated higher vaccine-type carriage prevalence at 18 months of age when compared to the full dose arm. These results align with a dose-response relationship across products: full dose PCV13, full dose PCV10 and 40% PCV13 contain at least 0.88ug of saccharide, whereas 20% PCV13, 20% PCV10 and 20%PCV10 contain less than 0.88ug (Table 1). A schedule of 2 full primary doses of PCV10 elicited non-inferior immunogenicity to 3 full primary doses of PCV10 among 6 of the 7 ST-specific responses that were assayed (all except ST23F) at 18 weeks of age. The 2p+1 arm had lower vaccine-type carriage prevalence than the 3p+0 arm at 18 months of age.

The Government of Kenya has announced it aims to fully self-finance its routine immunisation programme by 2030. In 2022, the country switched from the PCV10 produced by GlaxoSmithKline (currently US$3.05 per dose) to a lower cost alternative produced by Serum Institute of India (SII; currently US$2.00 per dose). Off-label use of a 3-dose schedule of 40%-PCV13 (US$ 1.1 per dose) represents a more affordable option currently and could reduce the annual cost of purchasing PCV for an annual birth cohort of 1.5 million children, from 9 to 5 million USD (at the current costs, assuming no vaccine wastage). Furthermore, 4-dose vials of PCV13 contain a preservative, which enables multi-dose vials to be used for up to 28 days following first puncture. It would be feasible, to implement a 40% dose policy immediately by re-classifying the present 4-dose vials of PCV13 as a 10-dose vials of 40%-doses (0.2ml).

The SII PCV10 contains similar doses of saccharide as PCV13; however, because of differences in the manufacturing processes, we cannot assume our findings are generalisable to the SII PCV10. At the time of trial design, only the GSK PCV10 and PCV13 were available and we could not study how the SII PCV10, nor the newer 15 and 20 valent PCVs, performed at fractional doses.

At both immunogenicity timepoints, we used non-inferiority criteria that are used routinely in vaccine licensure studies. However, the endpoints were serologic, not clinical, endpoints. There is some evidence that the threshold of protection against IPD varies by serotype^41^, and that correlates of protection against carriage are substantially higher than those against invasive disease^42^. It is unclear whether the lower, albeit non-inferior, immunogenicity of 40% PCV13, would influence protection against carriage acquisition or against pneumococcal disease. We did not detect increased vaccine-serotype carriage prevalence during our study; however, our trial population was under substantial indirect protection conferred by the high coverage of PCV10 in the routine immunisation system. Furthermore, it is unknown how a 40% dose will impact durability of immunity, follow-up over several years will reveal whether the rate of antibody waning over time differs by arm.

None of the children enrolled on the study acquired HIV, although a small number were exposed to the virus. The study was not designed to determine whether HIV-infected infants would mount a protective response following a schedule of 40%-PCV13. In Kenya, it is estimated that 0.9% of infants have HIV^43^.

In conclusion, 40% doses of PCV13 in a 2p+1 schedule generated non-inferior immune responses and no differences in vaccine type carriage prevalence when compared to a full dose schedule. Although the long-term impact of switching to a 40% PCV13 schedule on duration of immunity and carriage transmission is unclear and the findings cannot be generalised to HIV+ populations, an off-label, 3-dose schedule using 40% PCV13 is a less costly alternative to a full dose PCV programme in Gavi-transitioning LMICs and middle-income countries unsupported by Gavi.

## Supplementary Material

supplement

## Figures and Tables

**Table 1:** The available vaccine formulations and proposed fractional (20% and 40%) dose of PCV10 (GSK) and PCV13 (Pfizer)

Serotype-specific Saccharide dose (μg)	1	3	4	5	6A	6B	7F	9V	14	18C	19A	19F	23F
**PCV13** ^1^	2.2	2.2	2.2	2.2	2.2	4.4	2.2	2.2	2.2	2.2	2.2	2.2	2.2
**40%-PCV13**	0.88	0.88	0.88	0.88	0.88	1.76	0.88	0.88	0.88	0.88	0.88	0.88	0.88
**20%-PCV13**	0.44	0.44	0.44	0.44	0.44	0.88	0.44	0.44	0.44	0.44	0.44	0.44	0.44
**PCV10** ^2^	1.0		3.0	1.0		1.0	1.0	1.0	1.0	3.0		3.0	1.0
**40%-PCV10**	0.40		1.2	0.40		0.40	0.40	0.40	0.40	1.20		1.20	0.40
**20%-PCV10**	0.2		0.6	0.2		0.2	0.2	0.2	0.2	0.6		0.6	0.2

1 The saccharide in PCV13 is conjugated to CRM197 carrier protein

2 The saccharide in PCV10 is conjugated to Non-Typeable *H. influenzae* protein D, or tetanus toxoid (ST18C), or Diphtheria Toxin (ST19F).

**Table 2:** Carriage prevalence at 9 & 18 months of age

9-months of age			PCV13 2p+1						PCV10 2p+1				PCV10 3+0	
	Full dose		40% dose		20% dose		Full dose		40% dose		20% dose		Full dose	
	N=207		N=210		N=206		N=198		N=203		N=209		N=206	
Serotype	n	%	n	%	n	%	n	%	n	%	n	%	n	%
PCV13 VTs	37	17.9	49	23.3	43	20.9	49	24.7	49	24.1	61	29.2	52	25.2
PCV10 VTs	10	4.8	16	7.6	13	6.3	10	5.1	12	5.9	18	8.6	9	4.4
3/6A/19A	27	13.0	33	15.7	30	14.6	39	19.7	37	18.2	43	20.6	43	20.9
6A/19A	20	9.7	28	13.3	25	12.1	33	16.7	26	12.8	38	18.2	35	17.0
Any Carriage	174	84.1	173	82.4	168	81.6	158	79.8	167	82.3	178	85.2	173	84.0
**18-months** **of age**			**PCV13 2p+1**						**PCV10 2p+1**				**PCV10 3+0**	
	**Full dose**		**40% dose**		**20% dose**		**Full dose**		**40% dose**		**20% dose**		**Full dose**	
	**N=193**		**N=196**		**N=191**		**N=179**		**N=194**		**N=190**		**N=221**	
**Serotype**	**n**	**%**	**n**	**%**	**n**	**%**	**n**	**%**	**n**	**%**	**n**	**%**	**n**	**%**
PCV13 VTs	34	17.6	37	18.9	31	16.2	34	19.0	53	27.3	52	27.4	52	23.5
PCV10 VTs	10	5.2	17	8.7	13	6.8	6	3.4	11	5.7	20	10.5	21	9.5
3/6A/19A	24	12.4	20	10.2	18	9.4	28	15.6	42	21.6	32	16.8	31	14.0
6A/19A	19	9.8	13	6.6	10	5.2	22	12.3	32	16.5	23	12.1	20	9.0
Any Carriage	149	77.2	149	76.0	129	67.5	133	74.3	140	72.2	137	72.1	160	72.4

Abbreviations: PCV: Pneumococcal conjugate vaccine; VTs: vaccine serotypes
