# Supplementary material for: Fractional Doses of Pneumococcal Conjugate Vaccine: A Non-Inferiority Trial
Source: N Engl J Med. Author manuscript; Available in PMC 2024 Oct 16. (PMC7616702; doi:10.1056/NEJMoa2314620)
Supplement: supplement [file EMS197591-supplement-supplement.pdf]

Fractional Doses of Pneumococcal Conjugate Vaccine: A Non-Inferiority Trial

Katherine E. Gallagher PhD, Ruth Lucinde MSc, Christian Bottomley PhD, Mary Kaniu MSc, Badaud Suaad MD, Mary Mutahi BSc, Laura Mwalekwa BSc, Sarah Ragab BSc, Louise Twi-Yeboah BSc, James A. Berkley FRCPCH, Mainga Hamaluba PhD, Angela Karani BSc, Jimmy Shangala MSc, Mark Otiende MSc, Elizabeth Gardiner BSc, Daisy Mugo BSc, Peter G. Smith DSc, Collins Tabu MSc, Fred Were MD, David Goldblatt MD PhD, J. Anthony G. Scott FMedSci.

Supplementary Tables

Contents

Table S1A: Exclusions from the post-primary series (18-week of age) per-protocol population (immunogenicity) 2

Table S1B: Exclusions from the post-boost (approx. 10-month of age) per-protocol population (immunogenicity) 3

Table S1C: Exclusions from the 9-month of age per-protocol population (carriage) 4

Table S1D. Exclusions from the 18-month of age per-protocol population (carriage) 5

Table S2A: Baseline characteristics of the post-primary (18-week of age) per-protocol cohort (n, %) 7

Table S2B. Baseline characteristics of the post-primary (18-week of age) per-protocol cohort (median, inter-quartile range) 8

Table S2C: Characteristics of the post-boost (10 months of age) per-protocol cohort (n, %) 9

Table S2D: Characteristics of the post-boost per-protocol cohort (median, inter-quartile range) 11

Table S3A: Percentage of vaccine responders (IgG concentration >=0.35 mcg/ml) post-primary series (18 weeks of age) 13

Table S3B: Comparison of vaccine responders between study arms post-primary series (18 weeks of age; RD, 95%CI) 14

Table S3C: Comparison of vaccine responders between study arms post-primary series (18 weeks of age; RD, 90%CI as per SAP) 15

Table S4A: Geometric mean IgG concentrations post-boost (approx. 10 months of age) 16

Table S4B: Comparison of geometric mean antibody concentrations post-boost between study arms (approx. 10 months of age; GMC Ratio, 95%CI) 17

Table S4C: Comparison of geometric mean antibody concentrations post-boost between study arms (approx. 10 months of age; Ratio, 90%CI as per SAP) 18

Table S4D: Geometric mean titers of opsonophagocytic activity (OPA) post-boost (approx. 10 months of age)[1] 19

Table S4E: Percentage of OPA titres >8 post-boost (approx. 10 months of age) 20

Table S4F: Comparison of responders (OPA titre >8) between study arms post-boost (approx. 10 months of age; RD, 95%CI) 21

Table S5A: Serotype-specific carriage prevalence at 9 months of age 22

Table S5B: Serotype-specific carriage prevalence at 18 months of age 23

Table S5C: Differences between study arms in carriage at 9 months of age (RD, 95%CI) 24

Table S5D: Differences between study arms in carriage at 18 months of age (RD, 95%CI) 24

Table S6A: Number (%) children who experienced a post-vaccination reaction after one or more doses of PCV 25

Table S6B: Serious Adverse Events (SAEs), Severe pneumonia SAEs, and adverse events, by study arm 27

Table S1A: Exclusions from the post-primary series (18-week of age) per-protocol population (immunogenicity)

	PCV13 2p+1			PCV10 2p+1			PCV10 3+0	All
	Full dose	40% dose	20% dose	Full dose	40% dose	20% dose	Full dose	
Per-protocol cohort	233	221	223	219	221	225	230	1,572
Ineligible[1]	1	0	0	0	2	0	0	3
Randomisation error[2]	0	0	2	3	3	2	3	13
Received PCV dose outside study[3]	31	39	26	29	38	33	22	218
2nd PCV dose missed or not in window	12	14	16	21	15	17	12	107
Withdrew from study	6	6	6	4	5	2	5	34
Died	1	0	0	1	0	0	0	2
18-week visit missed or not in window	12	13	20	11	11	15	21	103
Sample stored >48 hrs after collection[4]	0	0	1	0	0	0	0	1
Sample missing[5]	4	6	5	12	4	6	7	44
Total	300	299	299	300	299	300	300	2,097

Table S1B: Exclusions from the post-boost (approx. 10-month of age) per-protocol population (immunogenicity)

	PCV13 2p+1			PCV10 2p+1			PCV10 3+0	All
	Full dose	40% dose	20% dose	Full dose	40% dose	20% dose	Full dose	
PP cohort	190	188	189	183	188	193	0	1,131
Ineligible[1]	1	0	0	0	2	0	0	3
Randomisation error[2]	0	0	2	3	3	2	0	10
Received PCV dose outside study[3]	31	39	26	29	38	33	0	196
2nd PCV dose missed or not in window	12	14	16	21	15	17	0	95
3rd PCV dose missed or not in window[6]	39	28	39	44	33	34	0	217
Withdrew from study	8	7	8	4	5	3	0	35
Died	1	1	1	1	0	2	0	6
10-month visit missed or not in window	14	13	12	13	11	12	0	75
Sample stored >48hrs after collection[4]	0	0	0	1	0	0	0	1
Sample missing[5]	4	9	6	1	4	4	0	28
Total	300	299	299	300	299	300	0	1,797

Table S1C: Exclusions from the 9-month of age per-protocol population (carriage)

	PCV13 2p+1			PCV10 2p+1			PCV10 3+0	
	**Full dose**	**40% dose**	**20% dose**	**Full dose**	**40% dose**	**20% dose**	**Full dose**	**All**
Per-protocol cohort	207	210	206	198	203	209	206	1,439
Ineligible[1]	1	0	0	0	2	0	0	3
Randomisation error[2]	0	0	2	3	3	2	3	13
Received PCV dose outside study[3]	31	39	26	29	38	33	22	218
2nd PCV dose missed or not in window	12	14	16	21	15	17	10	105
3rd PCV dose missed or not in window[6]	39	28	39	44	33	34	6	223
Withdrew from study	8	7	8	4	5	3	3	38
Died	1	1	1	1	0	2	0	6
NPS sample missing	0	0	0	0	0	0	13	13
NPS collection out of window	1	0	1	0	0	0	37	39
Total	300	299	299	300	299	300	300	2,097

Table S1D. Exclusions from the 18-month of age per-protocol population (carriage)

	PCV13 2p+1			PCV10 2p+1			PCV10 3+0	
	**Full dose**	**40% dose**	**20% dose**	**Full dose**	**40% dose**	**20% dose**	**Full dose**	**All**
Per-protocol cohort	193	196	191	179	194	190	221	1,364
Ineligible[1]	1	0	0	0	2	0	0	3
Randomisation error[2]	0	0	2	3	3	2	3	13
Received PCV dose outside study[3]	31	39	26	29	38	33	22	218
2nd PCV dose missed or not in window	12	14	16	21	15	17	10	105
3rd PCV dose missed or not in window[6]	39	28	39	44	33	34	6	223
Withdrew from study	8	7	8	4	5	3	6	41
Died	1	1	1	1	0	2	0	6
NPS sample missing	0	0	0	0	0	2	0	2
NPS collection out of window	15	14	16	19	9	17	32	122
Total	300	299	299	300	299	300	300	2,097

Table 1A, 1B, 1C, 1D notes:

[1] Three participants were age-ineligible at enrolment (<6 weeks) and this was only checked and determined post-randomisation. Protocol violations were submitted for the incorrect enrolment and randomisation of these participants. An additional three participants were enrolled however were ineligible with a fever on physical examination and were not randomized – these participants therefore do not feature in this table, explaining the total of 2097.

[2] Randomisation/ vaccination errors occurred when staff identified the incorrect randomisation code for the participant and the participant received a product or dose that was not allocated. Protocol violations were submitted for these deviations from SOPs.

[3] Study procedures and follow-up had to pause between 20th March 2020 and 21st September 2020 due to the COVID-19 pandemic restrictions on working. During this time, we advised participants who had not completed their primary series of vaccinations to attend their local vaccination clinic to receive a full dose of PCV, as it was unclear when we would be able to access them to complete their primary series. This led to 205 participants of the 637 enrolled before 20th March 2020 receiving a *full* second dose of PCV rather than their allocated dose. A further 13 participants of the 1463 participants enrolled after the pandemic restrictions had been lifted, received a full second dose of PCV at a different vaccination clinic rather than attending the study clinic. There were measures in place to prevent this happening including: stapling a research participation ID card to the mother-child handbook, placing a sticker stating 'do not vaccinate' on the PCV vaccination record page with a contact phone number, and regular telephone calls to mothers to remind them to come back to the study clinic for their vaccinations rather than go elsewhere. At each visit, a staff member would ask the mother whether their child had received any injections since they saw the study nurse and check the mother-child booklet for any indication of a vaccine dose from another vaccination clinic. If there was any uncertainty e.g., the mother reported attending another clinic and getting a vaccine but a dose was not documented in the MCH book, a staff member visited the clinic the mother reported attending and sought the clinic's vaccination register book to confirm if a PCV dose was administered to the study participant. These 13 deviations were identified in this way either by report, documentation or follow up at neighbouring clinics.

[4] Two protocol violations were submitted for an incident whereby inadequate hand-over between lab staff led to one V4 and one V6 sample being misplaced and only stored >48 hours after sample collection.

[5] In some cases it was not possible to take a blood sample due to difficulty in locating an adequate vein, or due to parental refusal, these were submitted as protocol deviations.

[6] During the COVID-19 pandemic the window for the boost (3rd dose) visit was widened to 9-12 months of age; however, the pandemic still had a significant impact on attendance of the boost dose visit within-window: 249 of the 637 (39%) enrolled before the pandemic either missed their boost dose or attended the visit out-of-window, in-comparison to 226 of the 1463 (16%) participants enrolled after the COVID-19 restrictions had been lifted. The PCV10 3p+0 arm was excluded from the per-protocol population at 10-months of age as participants did not receive a boost dose as per the allocated schedule.

Table S2A: Baseline characteristics of the post-primary (18-week of age) per-protocol cohort (n, %)

	PCV13 2p+1						PCV10 2p+1						PCV10 3+0	
	Full dose		40% dose		20% dose		Full dose		40% dose		20% dose		Full dose	
	n	%	n	%	n	%	n	%	n	%	n	%	n	%
Season														
Jan-Mar	23	10	25	11	22	10	25	11	23	10	25	11	24	10
Apr-Jun	66	28	67	30	66	30	66	30	62	28	67	30	62	27
Jul-Sep	65	28	53	24	56	25	60	27	68	31	60	27	64	28
Oct-Dec	79	34	76	34	79	35	68	31	68	31	73	32	80	35
Year														
2019	44	19	38	17	44	20	43	20	40	18	42	19	49	21
2020	4	2	4	2	5	2	3	1	5	2	3	1	3	1
2021	185	79	179	81	174	78	173	79	176	80	180	80	178	77
Sex														
Male	116	50	104	47	117	52	114	52	107	48	106	47	118	51
Female	117	50	117	53	106	48	105	48	114	52	119	53	112	49
Infant HIV														
Positive	0	0	0	0	0	0	0	0	0	0	0	0	0	0
Negative	225	97	213	96	210	94	213	97	215	97	215	96	222	97
Unknown	8	3	8	4	13	6	6	3	6	3	10	4	8	3

Table S2B. Baseline characteristics of the post-primary (18-week of age) per-protocol cohort (median, inter-quartile range)

	PCV13 2p+1						PCV10 2p+1						PCV10 3+0	
	Full dose		40% dose		20% dose		Full dose		40% dose		20% dose		Full dose	
	Med.	IQR	Med.	IQR	Med.	IQR	Med.	IQR	Med.	IQR	Med.	IQR	Med.	IQR
Maternal age, yrs	25	21,31	26	21,31	26	21,32	25	21,30	27	21,31	26	22,31	27	22,32
Infant weight, kgs	4	4,5	4	4,5	5	4,5	4	4,5	5	4,5	4	4,5	4	4,5
Dose 1, age in days	43	42,45	43	42,46	44	42,46	44	42,46	43	42,45	43	42,45	44	42,46
Dose 2, age in days	101	99,103	101	99,104	100	99,103	100	98,103	100	98,102	100	98,103	72	70,74
Dose 3, age in days	277	273,290	276	273,284	276	273,286	277	274,286	276	273,289	277	273,290	101	99,104

Table S2C: Characteristics of the post-boost (10 months of age) per-protocol cohort (n, %)

	PCV13 2p+1						PCV10 2p+1					
	Full dose		40% dose		20% dose		Full dose		40% dose		20% dose	
	n	%	n	%	n	%	n	%	n	%	n	%
Season												
Jan-Mar	29	15	26	14	28	15	25	14	28	15	32	17
Apr-Jun	60	32	62	33	63	33	64	35	60	32	61	32
Jul-Sep	49	26	45	24	44	23	46	25	50	27	49	25
Oct-Dec	52	27	55	29	54	29	48	26	50	27	51	26
Year												
2019	11	6	10	5	8	4	10	5	8	4	10	5
2020	7	4	7	4	11	6	6	3	10	5	9	5
2021	172	91	171	91	170	90	167	91	170	90	174	90
Maternal HIV												
Positive	5	3	6	3	12	6	4	2	5	3	10	5
Negative	185	97	182	97	176	93	179	98	182	97	183	95
Unknown	0	0	0	0	1	1	0	0	1	1	0	0
Breastfeeding, M10												
No	1	1	2	1	4	2	1	1	0	0	1	1
Yes	188	99	186	99	185	98	181	99	188	100	192	99
Unknown	1	1	0	0	0	0	1	1	0	0	0	0
Sex												
Male	93	49	87	46	97	51	93	51	92	49	93	48

	PCV13 2p+1						PCV10 2p+1					
	Full dose		40% dose		20% dose		Full dose		40% dose		20% dose	
	n	%	n	%	n	%	n	%	n	%	n	%
Female	97	51	101	54	92	49	90	49	96	51	100	52
Infant HIV												
Positive	0	0	0	0	0	0	0	0	0	0	0	0
Negative	185	97	182	97	176	93	179	98	183	97	183	95
Unknown	5	3	6	3	13	7	4	2	5	3	10	5

Note: Unless otherwise stated characteristics are those recorded at enrolment

Table S2D: Characteristics of the post-boost per-protocol cohort (median, inter-quartile range)

	PCV13 2p+1						PCV10 2p+1					
	Full dose		40% dose		20% dose		Full dose		40% dose		20% dose	
	Med.	IQR	Med.	IQR	Med.	IQR	Med.	IQR	Med.	IQR	Med.	IQR
Maternal age, yrs	24	21,30	26	21,32	26	21,32	25	21,30	26	21,31	26	22,30
Infant weight, kgs	4	4,5	4	4,5	5	4,5	4	4,5	4	4,5	4	4,5
Dose 1, age in days	43	42,45	43	42,46	43	42,46	43	42,45	43	42,45	43	42,45
Dose 2, age in days	100	99,103	100	99,104	100	98,103	100	98,102	100	98,102	100	98,102
Dose 3, age in days	277	273,283	275	272,281	275	273,283	276	273,282	276	273,282	276	273,283

Table S2E: The representativeness of the study population

Characteristic	Study Population at 10 months of age		KHDSS surveillance population (2021)[1]	
	**n**	**%**	**n**	**%**
All	1131		307,877	
Ethnicity				
Giriama	836	73.9	141,150	45.9
Chonyi	49	4.3	90,067	29.3
Kauma	56	5.0	32,699	10.6
Kikuyu	5	0.4	2,000	0.7
Kamba	26	2.3	3,199	1.0
Luo	16	1.4	2,703	0.9
Arab	0	0.0	1,261	0.4
Asian	2	0.2	80	0.0
Other[2]	141	12.5	67,417	21.9
Sex among 0-4 year olds[3]				
Male	555	49.1	22,002	50.8
Female	576	50.9	21,335	49.2

[1] The Kilifi Health and Demographic surveillance system (KHDSS) monitors a population of approximately 300,000 residents of Kilifi County, Kenya. No information on Race is collected; however, detailed information on ethnic group is presented. The study population was recruited from within the KHDSS and its surrounding areas.
[2] The 'Other' category is a mixture of 36 other Kenyan ethnic groups which each comprise 0-2% of the general population.
[3] The trial recruited infants between 6-8 weeks of age, this is the population invited to the routine immunisation programme for vaccinations

Table S3A: Percentage of vaccine responders (IgG concentration >=0.35 mcg/ml) post-primary series (18 weeks of age)

	PCV13 2p+1						PCV10 2p+1[1]						PCV10 3+0[2]	
	Full dose		40% dose		20% dose		Full dose		40% dose		20% dose		Full dose	
**Serotype**	**n/N**	**%**	**n/N**	**%**	**n/N**	**%**	**n/N**	**%**	**n/N**	**%**	**n/N**	**%**	**n/N**	**%**
1	232/232	100	220/221	100	221/222	100	214/218	98	216/221	98	212/224	95	0/0	
4	230/233	99	221/221	100	217/222	98	212/218	97	213/221	96	211/224	94	222/230	97
5	225/232	97	214/221	97	208/222	94	203/218	93	189/220	86	185/224	83	0/0	
6B	215/232	93	194/218	89	170/220	77	190/218	87	183/221	83	186/225	83	203/230	88
7F	232/232	100	220/221	100	221/222	100	216/218	99	214/221	97	218/224	97	0/0	
9V	226/233	97	215/221	97	213/223	96	211/219	96	210/221	95	205/225	91	223/230	97
14	223/232	96	206/221	93	199/222	90	211/218	97	213/221	96	215/225	96	225/229	98
18C	220/232	95	210/221	95	198/222	89	211/219	96	212/221	96	209/224	93	228/230	99
19F	231/231	100	219/219	100	221/221	100	215/217	99	212/213	100	217/222	98	226/230	98
23F	202/230	88	188/220	85	169/221	76	169/218	78	148/221	67	151/224	67	196/230	85
3	210/227	93	188/216	87	136/219	62	0/0		0/0		0/0		0/0	
6A	208/233	89	200/220	91	190/223	85	0/0		0/0		0/0		0/0	
19A	226/231	98	213/221	96	214/222	96	0/0		0/0		0/0		0/0	

Table 3A notes: [1] Samples from participants in the PCV10 2p+1 arms were assayed for IgG to the 10 PCV10 serotypes.
[2] Samples from the participants in the PCV10 3p+0 arm were assayed for IgG to 7 (PCV7) serotypes due to funding constraints for this secondary endpoint. The enrolment and sampling of the 3p+0 arm was funded through a small grant from the NIHR Mucosal Pathogens Research Unit.

Table S3B: Comparison of vaccine responders between study arms post-primary series (18 weeks of age; RD, 95%CI)

	PCV13				PCV10[1]					
	40% dose - Full dose		20% dose - Full dose		40% dose - Full dose		20% dose - Full dose		2p+1 - 3p+0	
**Serotype**	**RD**	**95% CI**	**RD**	**95% CI**	**RD**	**95% CI**	**RD**	**95% CI**	**RD**	**95% CI**
1	-0.5	-2.0, 1.0	-0.5	-2.0, 1.0	-0.4	-3.3, 2.5	-3.5	-7.1, 0.1		
4	1.3	-0.6, 3.1	-1.0	-3.7, 1.7	-0.9	-4.3, 2.6	-3.1	-6.9, 0.9	0.7	-2.7, 4.1
5	-0.2	-3.5, 3.2	-3.3	-7.3, 0.7	-7.2	-12.9, -1.4	-10.5	-16.5, -4.4		
6B	-3.7	-9.1, 1.7	-15.4	-21.8, -8.8	-4.4	-11.0, 2.4	-4.5	-11.1, 2.2	-1.1	-7.2, 5.0
7F	-0.5	-2.0, 1.0	-0.5	-2.0, 1.0	-2.2	-5.1, 0.7	-1.8	-4.5, 1.0		
9V	0.3	-3.0, 3.5	-1.5	-5.1, 2.2	-1.3	-5.3, 2.6	-5.2	-9.8,-0.6	-0.6	-4.1, 2.9
14	-2.9	-7.2, 1.4	-6.5	-11.2, -1.6	-0.4	-4.0, 3.2	-1.2	-4.9, 2.5	-1.5	-4.6, 1.6
18C	0.2	-4.0, 4.3	-5.6	-10.7, -0.5	-0.4	-4.2, 3.4	-3.0	-7.2, 1.2	-2.8	-5.8, 0.2
19F	0.0	-1.2, 1.2	0.0	-1.2, 1.2	0.5	-1.6, 2.4	-1.3	-3.9, 1.3	0.8	-1.6, 3.2
23F	-2.4	-8.7, 4.0	-11.4	-18.3, -4.2	-10.6	-18.8, -2.2	-10.1	-18.3, -1.8	-7.7	-14.8, -0.4
3	-5.5	-11.1, 0.3	-30.4	-37.4,-22.9						
6A	1.6	-4.0, 7.2	-4.1	-10.2, 2.1						
19A	-1.5	-4.7, 1.8	-1.4	-4.7, 1.8						

[1] Samples from participants in the PCV10 2p+1 arms were assayed for IgG to the 10 PCV10 serotypes. Samples from the participants in the PCV10 3p+0 arm were assayed for IgG to 7 (PCV7) serotypes due to funding constraints for this secondary endpoint. The enrolment and sampling of the 3p+0 arm was funded through a small grant from the NIHR Mucosal Pathogens Research Unit.

Table S3C: Comparison of vaccine responders between study arms post-primary series (18 weeks of age; RD, 90%CI as per SAP)

	PCV13				PCV10					
	40% dose - Full dose		20% dose - Full dose		40% dose - Full dose[1]		20% dose - Full dose[1]		2p+1 - 3p+0[2]	
Serotype	RD	90% CI	RD	90% CI	RD	90% CI	RD	90% CI	RD	90% CI
1	-0.5	-1.7, 0.8	-0.5	-1.7, 0.8	-0.4	-2.8, 2.0	-3.5	-6.5, -0.4		
4	1.3	-0.3, 2.8	-1.0	-3.2, 1.3	-0.9	-3.8, 2.1	-3.1	-6.3, 0.3	0.7	-2.2, 3.6
5	-0.2	-3.0, 2.7	-3.3	-6.6, 0.1	-7.2	-12.0, -2.3	-10.5	-15.5, -5.4		
6B	-3.7	-8.2, 0.9	-15.4	-20.7, -9.8	-4.4	-9.9, 1.3	-4.5	-10.0, 1.2	-1.1	-6.3, 4.0
7F	-0.5	-1.7, 0.8	-0.5	-1.7, 0.8	-2.2	-4.6, 0.2	-1.8	-4.0, 0.6		
9V	0.3	-2.5, 3.0	-1.5	-4.5, 1.6	-1.3	-4.6, 2.0	-5.2	-9.0, -1.3	-0.6	-3.6, 2.3
14	-2.9	-6.5, 0.7	-6.5	-10.5, -2.4	-0.4	-3.4, 2.6	-1.2	-4.3, 1.9	-1.5	-4.1, 1.1
18C	0.2	-3.3, 3.7	-5.6	-9.9,-1.4	-0.4	-3.6, 2.7	-3.0	-6.6, 0.6	-2.8	-5.3, -0.3
19F	0.0	-1.0, 1.0	0.0	-1.0, 1.0	0.5	-1.2, 2.1	-1.3	-3.5, 0.9	0.8	-1.2, 2.8
23F	-2.4	-7.7, 2.9	-11.4	-17.2, -5.4	-10.6	-17.4, -3.5	-10.1	-16.9, -3.1	-7.7	-13.7, -1.6
3	-5.5	-10.2, -0.7	-30.4	-36.3,-24.0						
6A	1.6	-3.1, 6.3	-4.1	-9.2, 1.1						
19A	-1.5	-4.2, 1.3	-1.4	-4.2, 1.3						

Table notes: The approved SAP was to use a 1-sided 95%CI (i.e. a 2-sided 90%CI); therefore this table is to document the analyses that were pre-specified in the SAP. The manuscript reports a more stringent analysis as the primary analysis which uses 2-sided 95%CI, this aligns with what is used in licensure.

[1] Samples from participants in the PCV10 2p+1 arms were assayed for IgG to the 10 PCV10 serotypes.

[2] Samples from the participants in the PCV10 3p+0 arm were assayed for IgG to 7 (PCV7) serotypes due to funding constraints for this secondary endpoint. The enrolment and sampling of the 3p+0 arm was funded through a small grant from the NIHR Mucosal Pathogens Research Unit.

Table S4A: Geometric mean IgG concentrations post-boost (approx. 10 months of age)

	PCV13 2p+1						PCV10 2p+1					
	Full dose		40% dose		20% dose		Full dose		40% dose		20% dose	
**Serotype**	**N**	**GMC**	**N**	**GMC**	**N**	**GMC**	**N**	**GMC**	**N**	**GMC**	**N**	**GMC**
1	190	4.4	188	3.4	189	2.6	180	3.1	180	1.7	182	1.3
4	190	3.3	188	2.3	189	1.9	183	2.8	188	2.0	191	1.5
5	190	1.9	188	1.5	189	1.3	183	0.8	187	0.5	192	0.4
6B	190	12.9	188	11.2	189	7.5	183	4.5	188	3.0	193	2.3
7F	190	7.2	188	5.3	189	4.3	183	3.5	188	2.3	193	1.7
9V	189	4.0	188	3.0	187	2.7	181	2.8	186	1.9	188	1.4
14	190	7.6	188	6.3	189	4.3	183	4.5	188	2.7	193	1.8
18C	190	3.6	188	2.5	189	1.8	183	7.8	188	4.5	192	3.2
19F	190	13.1	186	10.6	188	9.8	180	14.8	184	12.2	188	9.5
23F	189	4.9	186	4.0	187	3.0	174	1.7	185	1.0	186	0.7
3	186	1.0	187	0.8	189	0.7	0		0		0	
6A	190	9.9	188	7.0	189	5.7	0		0		0	
19A	187	9.3	184	7.5	188	6.1	0		0		0	

Table S4B: Comparison of geometric mean antibody concentrations post-boost between study arms (approx. 10 months of age; GMC Ratio, 95%CI)

	PCV13				PCV10			
	40% dose / Full dose		20% dose / Full dose		40% dose / Full dose		20% dose / Full dose	
**Serotype**	**Ratio**	**95% CI**	**Ratio**	**95% CI**	**Ratio**	**95% CI**	**Ratio**	**95% CI**
1	0.76	0.65,0.89	0.59	0.50,0.69	0.57	0.47,0.68	0.42	0.35,0.51
4	0.72	0.61,0.84	0.58	0.50,0.67	0.70	0.61,0.81	0.53	0.46,0.62
5	0.77	0.66,0.89	0.66	0.57,0.76	0.69	0.61,0.79	0.54	0.47,0.61
6B	0.87	0.74,1.02	0.58	0.49,0.70	0.65	0.57,0.75	0.51	0.45,0.59
7F	0.73	0.64,0.84	0.60	0.52,0.68	0.66	0.58,0.76	0.48	0.41,0.55
9V	0.76	0.65,0.89	0.68	0.58,0.79	0.66	0.58,0.76	0.49	0.42,0.57
14	0.83	0.67,1.04	0.57	0.45,0.71	0.59	0.46,0.75	0.40	0.32,0.52
18C	0.69	0.59,0.81	0.50	0.43,0.58	0.58	0.49,0.69	0.42	0.35,0.50
19F	0.81	0.68,0.96	0.75	0.63,0.89	0.83	0.68,1.00	0.64	0.53,0.78
23F	0.80	0.66,0.98	0.61	0.49,0.75	0.58	0.49,0.70	0.41	0.34,0.49
3	0.84	0.72,0.99	0.76	0.64,0.91				
6A	0.71	0.59,0.85	0.57	0.48,0.69				
19A	0.81	0.67,0.97	0.66	0.54,0.80				

Table S4C: Comparison of geometric mean antibody concentrations post-boost between study arms (approx. 10 months of age; Ratio, 90%CI as per SAP)

	PCV13				PCV10			
	40% dose / Full dose		20% dose / Full dose		40% dose / Full dose		20% dose / Full dose	
**Serotype**	**Ratio**	**90% CI**	**Ratio**	**90% CI**	**Ratio**	**90% CI**	**Ratio**	**90% CI**
1	0.76	0.67,0.87	0.59	0.52,0.67	0.57	0.48,0.66	0.42	0.36,0.50
4	0.72	0.63,0.82	0.58	0.51,0.65	0.70	0.62,0.79	0.53	0.47,0.61
5	0.77	0.68,0.87	0.66	0.58,0.74	0.69	0.62,0.78	0.54	0.48,0.60
6B	0.87	0.76,0.99	0.58	0.51,0.68	0.65	0.58,0.74	0.51	0.46,0.58
7F	0.73	0.66,0.82	0.60	0.54,0.66	0.66	0.59,0.75	0.48	0.42,0.54
9V	0.76	0.67,0.87	0.68	0.60,0.77	0.66	0.59,0.74	0.49	0.43,0.55
14	0.83	0.69,1.00	0.57	0.47,0.69	0.59	0.48,0.72	0.40	0.33,0.50
18C	0.69	0.60,0.79	0.50	0.44,0.57	0.58	0.50,0.67	0.42	0.36,0.48
19F	0.81	0.70,0.93	0.75	0.64,0.87	0.83	0.70,0.97	0.64	0.55,0.76
23F	0.80	0.68,0.95	0.61	0.51,0.72	0.58	0.50,0.68	0.41	0.35,0.48
3	0.84	0.74,0.97	0.76	0.66,0.88				
6A	0.71	0.61,0.82	0.57	0.49,0.67				
19A	0.81	0.69,0.94	0.66	0.56,0.78				

Table notes: The approved SAP was to use a 1-sided 95%CI (i.e. a 2-sided 90%CI); therefore this table is to document the results of the analyses that were pre-specified in the SAP. The manuscript reports a more stringent analysis as the primary analysis which uses 2-sided 95%CI, this aligns with what is used in licensure and is described in the methods.

Table S4D: Geometric mean titers of opsonophagocytic activity (OPA) post-boost (approx. 10 months of age)[1]

	PCV13 2p+1						PCV10 2p+1					
	Full dose		40% dose		20% dose		Full dose		40% dose		20% dose	
Serotype	N	GMT	N	GMT	N	GMT	N	GMT	N	GMT	N	GMT
1	48	266	50	217	49	204	45	271	49	147	48	120
4	49	1,821	48	1,640	50	1,784	45	1,361	47	857	47	602
5	49	496	50	387	50	392	48	461	48	313	49	213
6B	49	2,749	50	2,898	50	2,103	48	1,007	47	610	49	481
7F	49	4,112	50	2,788	50	2,938	47	2,183	49	1,482	49	1,153
9V	49	1,448	50	1,105	50	735	48	656	49	338	49	262
14	49	1,383	49	859	50	1,359	46	1,069	48	430	49	270
18C	49	1,325	50	1,131	50	872	48	3,584	49	2,164	49	1,777
19F	49	1,037	50	842	50	732	47	1,182	49	972	49	446
23F	49	3,047	50	3,706	50	3,541	48	797	49	340	46	336

[1] Assays of opsonophagocytic activity were performed on a random selection of 50 samples of sera from the post-boost timepoint

Table S4E: Percentage of OPA titres >8 post-boost (approx. 10 months of age)

	PCV13 2p+1						PCV10 2p+1					
	Full dose		40% dose		20% dose		Full dose		40% dose		20% dose	
**Serotype**	**n/N**	**%**	**n/N**	**%**	**n/N**	**%**	**n/N**	**%**	**n/N**	**%**	**n/N**	**%**
1	48/48	100	49/50	98	47/49	96	44/45	98	46/49	94	44/48	92
4	48/49	98	48/48	100	50/50	100	45/45	100	47/47	100	46/47	98
5	49/49	100	50/50	100	50/50	100	47/48	98	47/48	98	48/49	98
6B	49/49	100	50/50	100	49/50	98	48/48	100	47/47	100	48/49	98
7F	49/49	100	50/50	100	50/50	100	47/47	100	49/49	100	49/49	100
9V	49/49	100	50/50	100	50/50	100	48/48	100	49/49	100	48/49	98
14	47/49	96	44/49	90	49/50	98	46/46	100	42/48	88	41/49	84
18C	49/49	100	50/50	100	50/50	100	48/48	100	49/49	100	49/49	100
19F	49/49	100	49/50	98	48/50	96	44/47	94	48/49	98	43/49	88
23F	49/49	100	50/50	100	50/50	100	48/48	100	49/49	100	46/46	100

Table S4F: Comparison of responders (OPA titre >8) between study arms post-boost (approx. 10 months of age; RD, 95%CI)

	PCV13				PCV10			
	40% dose - Full dose		20% dose - Full dose		40% dose - Full dose		20% dose - Full dose	
**Serotype**	**RD**	**95% CI**	**RD**	**95% CI**	**RD**	**95% CI**	**RD**	**95% CI**
1	-2.0	-8.4, 4.7	-4.1	-11.4, 3.7	-3.9	-13.0, 5.8	-6.1	-15.9, 4.4
4	2.0	-4.7, 8.5	2.0	-4.5, 8.5	0.0	-5.6, 5.8	-2.1	-8.9, 5.0
5	0.0	-5.3, 5.4	0.0	-5.3, 5.4	0.0	-7.7, 7.7	0.0	-7.5, 7.7
6B	0.0	-5.3, 5.4	-2.0	-8.4, 4.6	0.0	-5.6, 5.5	-2.0	-8.5, 4.7
7F	0.0	-5.3, 5.4	0.0	-5.3, 5.4	0.0	-5.4, 5.6	0.0	-5.4, 5.6
9V	0.0	-5.3, 5.4	0.0	-5.3, 5.4	0.0	-5.4, 5.5	-2.0	-8.5, 4.7
14	-6.1	-16.8, 5.1	2.1	-6.3,10.3	-12.5	-22.3, -1.5	-16.3	-26.8, -4.3
18C	0.0	-5.3, 5.4	0.0	-5.3, 5.4	0.0	-5.4, 5.5	0.0	-5.4, 5.5
19F	-2.0	-8.4, 4.6	-4.0	-11.2, 3.6	4.3	-5.1,13.6	-5.9	-17.7, 6.6
23F	0.0	-5.3, 5.4	0.0	-5.3, 5.4	0.0	-5.4, 5.5	0.0	-5.7, 5.5

Table S5A: Serotype-specific carriage prevalence at 9 months of age

	PCV13 2p+1						PCV10 2p+1						PCV10 3+0	
	Full dose		40% dose		20% dose		Full dose		40% dose		20% dose		Full dose	
	N=207		N=210		N=206		N=198		N=203		N=209		N=206	
**Serotype**	**n**	**%**	**n**	**%**	**n**	**%**	**n**	**%**	**n**	**%**	**n**	**%**	**n**	**%**
1	0	0.0	0	0.0	0	0.0	0	0.0	0	0.0	0	0.0	0	0.0
4	0	0.0	1	0.5	0	0.0	0	0.0	0	0.0	0	0.0	0	0.0
5	0	0.0	0	0.0	0	0.0	0	0.0	0	0.0	0	0.0	0	0.0
6B	0	0.0	0	0.0	0	0.0	0	0.0	0	0.0	1	0.5	0	0.0
7F	0	0.0	0	0.0	0	0.0	0	0.0	0	0.0	0	0.0	0	0.0
9V	0	0.0	0	0.0	1	0.5	0	0.0	0	0.0	0	0.0	0	0.0
14	0	0.0	3	1.4	3	1.5	3	1.5	3	1.5	2	1.0	4	1.9
18C	0	0.0	0	0.0	0	0.0	0	0.0	0	0.0	0	0.0	0	0.0
19F	10	4.8	10	4.8	8	3.9	7	3.5	8	3.9	11	5.3	3	1.5
23F	0	0.0	2	1.0	1	0.5	0	0.0	1	0.5	4	1.9	2	1.0
3	7	3.4	5	2.4	5	2.4	6	3.0	11	5.4	5	2.4	8	3.9
6A	10	4.8	17	8.1	12	5.8	16	8.1	13	6.4	22	10.5	19	9.2
19A	10	4.8	11	5.2	13	6.3	17	8.6	13	6.4	16	7.7	16	7.8
PCV13	37	17.9	49	23.3	43	20.9	49	24.7	49	24.1	61	29.2	52	25.2
PCV10	10	4.8	16	7.6	13	6.3	10	5.1	12	5.9	18	8.6	9	4.4
3/6A/19A	27	13.0	33	15.7	30	14.6	39	19.7	37	18.2	43	20.6	43	20.9
6A/19A	20	9.7	28	13.3	25	12.1	33	16.7	26	12.8	38	18.2	35	17.0
Any carriage	174	84.1	173	82.4	168	81.6	158	79.8	167	82.3	178	85.2	173	84.0

Table S5B: Serotype-specific carriage prevalence at 18 months of age

	PCV13 2p+1						PCV10 2p+1						PCV10 3+0	
	Full dose		40% dose		20% dose		Full dose		40% dose		20% dose		Full dose	
	N=193		N=196		N=191		N=179		N=194		N=190		N=221	
**Serotype**	**n**	**%**	**n**	**%**	**n**	**%**	**n**	**%**	**n**	**%**	**n**	**%**	**n**	**%**
1	0	0.0	0	0.0	0	0.0	0	0.0	0	0.0	1	0.5	0	0.0
4	0	0.0	1	0.5	0	0.0	0	0.0	0	0.0	0	0.0	0	0.0
5	0	0.0	0	0.0	0	0.0	0	0.0	0	0.0	0	0.0	0	0.0
6B	0	0.0	0	0.0	0	0.0	0	0.0	0	0.0	1	0.5	0	0.0
7F	0	0.0	0	0.0	0	0.0	0	0.0	0	0.0	0	0.0	0	0.0
9V	2	1.0	0	0.0	0	0.0	0	0.0	0	0.0	1	0.5	0	0.0
14[1]	1	0.5	0	0.0	2	1.0	1	0.6	4	2.1	10	5.3	6	2.7
18C	1	0.5	0	0.0	0	0.0	0	0.0	0	0.0	0	0.0	1	0.5
19F[2]	6	3.1	14	7.1	11	5.8	5	2.8	6	3.1	7	3.7	14	6.3
23F	0	0.0	2	1.0	0	0.0	0	0.0	1	0.5	0	0.0	0	0.0
3	5	2.6	7	3.6	8	4.2	6	3.4	10	5.2	9	4.7	11	5.0
6A	7	3.6	8	4.1	2	1.0	11	6.1	19	9.8	17	8.9	12	5.4
19A[3]	12	6.2	5	2.6	8	4.2	11	6.1	13	6.7	6	3.2	8	3.6
PCV13	34	17.6	37	18.9	31	16.2	34	19.0	53	27.3	52	27.4	52	23.5
PCV10[4]	10	5.2	17	8.7	13	6.8	6	3.4	11	5.7	20	10.5	21	9.5
3/6A/19A	24	12.4	20	10.2	18	9.4	28	15.6	42	21.6	32	16.8	31	14.0
6A/19A	19	9.8	13	6.6	10	5.2	22	12.3	32	16.5	23	12.1	20	9.0
Any carriage	149	77.2	149	76.0	129	67.5	133	74.3	140	72.2	137	72.1	160	72.4

Table S5C: Differences between study arms in carriage at 9 months of age (RD, 95%CI)

Comparison	Serotypes	RD	95% CI
PCV13 40% dose vs full	PCV13	5.5	-2.3,13.1
PCV13 20% dose vs full	PCV13	3.0	-4.7,10.6
PCV10 40% dose vs full	PCV10	0.9	-3.8, 5.4
PCV10 20% dose vs full	PCV10	3.6	-1.5, 8.5
PCV13 vs PCV10 (both full dose)	3/6A/19A	-6.7	-13.8, 0.6
PCV13 vs PCV10 (both full dose)	6A/19A	-7.0	-13.6, -0.3
PCV10 2p+1 vs 3+0 (both full dose)	PCV10	0.7	-3.6, 5.0

Table S5D: Differences between study arms in carriage at 18 months of age (RD, 95%CI)

Comparison	Serotypes	RD	95% CI
PCV13 40% dose vs full	PCV13	1.3	-6.4, 8.9
PCV13 20% dose vs full	PCV13	-1.4	-8.9, 6.2
PCV10 40% dose vs full	PCV10	2.3	-2.1, 6.6
PCV10 20% dose vs full	PCV10	7.2	1.8,12.3
PCV13 vs PCV10 (both full dose)	3/6A/19A	-3.2	-10.3, 3.9
PCV13 vs PCV10 (both full dose)	6A/19A	-2.4	-8.9, 4.0
PCV10 2p+1 vs 3+0 (both full dose)	PCV10	-6.2	-10.8, -1.2

Table S6A: Number (%) children who experienced a post-vaccination reaction after one or more doses of PCV

	PCV13 2p+1						PCV10 2p+1						PCV10 3+0		
	Full dose		40% dose		20% dose		Full dose		40% dose		20% dose		Full dose		
	n	%	n	%	n	%	n	%	n	%	n	%	n	%	p-value[1]
Rash															1.000
None	300	100.0	299	100.0	299	100.0	299	99.7	299	100.0	300	100.0	300	100.0	
Localised	0	0.0	0	0.0	0	0.0	1	0.3	0	0.0	0	0.0	0	0.0	
Irritability															0.645
None	299	99.7	296	99.0	298	99.7	295	98.3	296	99.0	297	99.0	298	99.3	
More than usual	1	0.3	3	1.0	1	0.3	5	1.7	3	1.0	3	1.0	2	0.7	
Drowsiness															1.000
None	300	100.0	299	100.0	299	100.0	299	99.7	299	100.0	299	99.7	300	100.0	
More than usual	0	0.0	0	0.0	0	0.0	1	0.3	0	0.0	1	0.3	0	0.0	
Loss of appetite															0.931
None	299	99.7	299	100.0	299	100.0	300	100.0	298	99.7	299	99.7	300	100.0	
Eats less than usual	1	0.3	0	0.0	0	0.0	0	0.0	1	0.3	1	0.3	0	0.0	
Pain															0.067
None	286	95.3	296	99.0	292	97.7	294	98.0	289	96.7	296	98.7	293	97.7	
Mild reaction to touch	14	4.7	3	1.0	7	2.3	6	2.0	8	2.7	3	1.0	7	2.3	

	PCV13 2p+1						PCV10 2p+1						PCV10 3+0		
	Full dose		40% dose		20% dose		Full dose		40% dose		20% dose		Full dose		
	n	%	n	%	n	%	n	%	n	%	n	%	n	%	p-value[1]
Cries on touch	0	0.0	0	0.0	0	0.0	0	0.0	2	0.7	1	0.3	0	0.0	
Redness															0.426
None	300	100.0	298	99.7	299	100.0	300	100.0	299	100.0	300	100.0	300	100.0	
<=2.5cm	0	0.0	0	0.0	0	0.0	0	0.0	0	0.0	0	0.0	0	0.0	
>2.5cm	0	0.0	1	0.3	0	0.0	0	0.0	0	0.0	0	0.0	0	0.0	
Swelling															
None	300	100.0	299	100.0	299	100.0	300	100.0	299	100.0	300	100.0	300	100.0	
<=2.5cm	0	0.0	0	0.0	0	0.0	0	0.0	0	0.0	0	0.0	0	0.0	

[1] P-values have not been adjusted for multiplicity

Table S6B: Serious Adverse Events (SAEs), Severe pneumonia SAEs, and adverse events, by study arm

	PCV13 2p+1						PCV10 2p+1						PCV10 3+0		
	Full dose		40% dose		20% dose		Full dose		40% dose		20% dose		Full dose		
	n	%	n	%	n	%	n	%	n	%	n	%	n	%	p-value
**SAE[1]**															0.69
None	293	98	291	97	285	95	293	98	293	98	291	97	292	97	
1	6	2	8	3	12	4	6	2	6	2	9	3	7	2	
2	1	0	0	0	2	1	1	0	0	0	0	0	0	0	
3	0	0	0	0	0	0	0	0	0	0	0	0	1	0	
Severe pneumonia SAE	3	1	5	2	7	2	6	2	4	1	6	2	9	3	
Severe pneumonia SAE with abnormal CXR	1	0	3	1	1	0	1	0	1	0	1	0	5	2	
Died															0.84
No	299	100	298	100	298	100	298	99	299	100	298	99	300	100	
Yes	1	0	1	0	1	0	2	1	0	0	2	1	0	0	
**All Respiratory AE[2]**															0.70
0	165	55	148	49	151	51	143	48	160	54	145	48	160	53	
1-3	109	36	123	41	123	41	134	45	117	39	130	43	110	37	
4-11	26	9	28	9	25	8	23	8	22	7	25	8	30	10	
Pneumonia AEs	8	3	16	5	8	3	9	3	5	2	7	2	8	3	

	PCV13 2p+1						PCV10 2p+1						PCV10 3+0		
	Full dose		40% dose		20% dose		Full dose		40% dose		20% dose		Full dose		
	n	%	n	%	n	%	n	%	n	%	n	%	n	%	p-value
Pneumonia AEs with abnormal CXR	1	0	4	1	4	1	2	1	1	0	0	0	2	1	

Abbreviations: AE: adverse event; CXR: chest x-ray; SAE: serious adverse event.
Table notes:

[1] Safety reports include 'initial reports' of events only. SAEs reported here are all SAEs. Severe pneumonia SAEs are a subcategory of all SAEs, severe pneumonia SAEs with abnormal chest x-ray are a further sub-category of severe pneumonia SAEs. Deaths were reported as SAEs. Severe pneumonia was defined using the WHO Pocket Book for Hospital care of Children (2013) i.e. cough or difficulty breathing/ fast breathing and some evidence of lower chest wall indrawing/ nasal flaring/ grunting (with or without other danger signs). Fast breathing was defined as $\geq$50 breaths per minute in 2-12 months olds and $\geq$40 breaths per minute is >12 months of age.

[2] In March 2020 a protocol amendment was approved to restrict reporting of AEs to only those affecting the respiratory tract to attempt to manage workload more effectively. Most of these were very minor coughs and colds, Pneumonia AEs were cases of non-severe pneumonia and are a subcategory of all AEs and underwent investigation as per SOPs with chest x-ray and a nasopharyngeal swab. Non-severe pneumonia cases were cases of cough or difficulty breathing without any other danger sign.
